# Disease resistance or growth: the role of plant hormones in balancing immune responses and fitness costs

**DOI:** 10.3389/fpls.2013.00155

**Published:** 2013-05-24

**Authors:** Nicolas Denancé, Andrea Sánchez-Vallet, Deborah Goffner, Antonio Molina

**Affiliations:** ^1^UMR 5546, Laboratoire de Recherche en Sciences Végétales, Université de ToulouseCastanet-Tolosan, France; ^2^UMR 5546, Laboratoire de Recherche en Sciences Végétales, Centre National de la Recherche ScientifiqueCastanet-Tolosan, France; ^3^Laboratory of Phytopathology, Wageningen UniversityWageningen, Netherlands; ^4^Centro de Biotecnología y Genómica de Plantas, Instituto Nacional de Investigación y Tecnología Agraria y Alimentaria, Universidad Politécnica de MadridPozuelo de Alarcón, Spain; ^5^Departamento de Biotecnología, Escuela Técnica Superior de Ingenieros Agrónomos, Universidad Politécnica de MadridMadrid, Spain

**Keywords:** abscisic acid, auxin, hormone crosstalk, pathogens, salicylic acid, trade-off, virulence factor

## Abstract

Plant growth and response to environmental cues are largely governed by phytohormones. The plant hormones ethylene, jasmonic acid, and salicylic acid (SA) play a central role in the regulation of plant immune responses. In addition, other plant hormones, such as auxins, abscisic acid (ABA), cytokinins, gibberellins, and brassinosteroids, that have been thoroughly described to regulate plant development and growth, have recently emerged as key regulators of plant immunity. Plant hormones interact in complex networks to balance the response to developmental and environmental cues and thus limiting defense-associated fitness costs. The molecular mechanisms that govern these hormonal networks are largely unknown. Moreover, hormone signaling pathways are targeted by pathogens to disturb and evade plant defense responses. In this review, we address novel insights on the regulatory roles of the ABA, SA, and auxin in plant resistance to pathogens and we describe the complex interactions among their signal transduction pathways. The strategies developed by pathogens to evade hormone-mediated defensive responses are also described. Based on these data we discuss how hormone signaling could be manipulated to improve the resistance of crops to pathogens.

## INTRODUCTION

In their natural environments, plants are under continuous biotic stress caused by different attackers (e.g., bacteria, fungi, viruses, oomycetes, and insects) that compromise plant survival and offspring. Given that green plants are the ultimate source of energy for most organisms, it is not surprising that plants have evolved a variety of resistance mechanisms that can be constitutively expressed or induced after pathogen or pest attack (Glazebrook, 2005; Panstruga et al., 2009). Plants have developed molecular mechanisms to detect pathogens and pests and to activate defense responses. The plant innate immune system relies in the specific detection by plant protein recognition receptors (PRRs) of relatively conserved molecules of the pathogen called pathogen-associated molecular patterns (PAMPs). This resistance response is known as PAMP-triggered immunity (PTI). Successful pathogens secrete effector proteins that deregulate PTI. To counteract this, plant resistance (R) proteins recognize effectors and activate effector-triggered immunity (ETI; reviewed in Dodds and Rathjen, 2010).

A fine-tune regulation of these immune responses is necessary because the use of metabolites in plant resistance may be detrimental to other physiological processes impacting negatively in other plant traits, such as biomass and seed production (Walters and Heil, 2007; Kempel et al., 2011). These physiological constrains, together with other factors such as the co-existence of plants with natural attackers, have contributed to drive the evolution of a dynamic and complex network system. Defense layers from separate cellular components and from diverse physiological processes are interconnected to reduce the inherent fitness cost of being well-defended (Chisholm et al., 2006; Panstruga et al., 2009; Schulze-Lefert and Panstruga, 2011). The resistance response is regulated by phytohormones, that are small molecules which synergistically and/or antagonistically work in a complex network to regulate many aspects of plant growth, development, reproduction, and response to environmental cues (Pieterse et al., 2009; Santner et al., 2009; Jaillais and Chory, 2010). Recent progresses have been made in understanding the complex hormone network that governs plant immunity, giving rise to a database containing information of the hormone-regulated genes (e.g., in *Arabidopsis thaliana*) and the phenotypic description of hormone-related mutants (Peng et al., 2009). In parallel, it has been found that pathogens have developed sophisticated molecular mechanisms to deregulate the biosynthesis of hormones and/or to interfere with hormonal signaling pathways, thus, facilitating the overcoming of plant defense mechanisms (Jones and Dangl, 2006; Dodds and Rathjen, 2010). The essential roles of salicylic acid (SA) and ethylene (ET)/jasmonic acid (JA)-mediated signaling pathways in resistance to pathogens are well described (Robert-Seilaniantz et al., 2011a). SA signaling positively regulates plant defense against biotrophic pathogens, that need alive tissue to complete their life cycle, whereas ET/JA pathways are commonly required for resistance to necrotrophic pathogens, that degrade plant tissue during infection, and to herbivorous pests (Glazebrook, 2005; Bari and Jones, 2009). Several exceptions for this general rule have been described, and thus SA pathway is also required for plant resistance to particular necrotrophic pathogens, whereas ET/JA pathways were found to be essential for resistance to some biotrophic pathogens (Berrocal-Lobo et al., 2002; Robert-Seilaniantz et al., 2011a). Additionally, other hormones such as auxins and abscisic acid (ABA), originally described for their function in the regulation of plant growth processes and the response to abiotic stresses, have recently emerged as crucial players in plant–pathogen interactions (Mauch-Mani and Mauch, 2005; Kazan and Manners, 2009; Ton et al., 2009; Fu and Wang, 2011). All the phytohormone pathways are linked to each other in a huge, complex and still obscure network. For example, ET, ABA, auxin, gibberellins, and cytokinins pathways are considered as hormone modulators of the SA–JA signaling backbone (Pieterse et al., 2012).

To develop hormone-based breeding strategies aiming to improve crop resistance to pathogens, we need to understand the intricate regulation of hormone homeostasis during plant–pathogen interactions, and how pathogens interfere with this hormone regulation. Indeed, manipulation of a plant hormone pathway can result in enhanced resistance to a particular pathogen, but it could also have a strong negative effect on plant growth and resistance to a distinct type of pathogen with a different life style (Holeski et al., 2012). In this review, we will discuss novel insights on the complex role of phytohormones in balancing plant innate immunity and development, with a special focus on the regulatory crosstalk of auxins, SA, and ABA. We will also learn about decoy strategies employed by the attackers to disturb hormone-mediated defense responses in plants, and we will describe how misregulation of these hormone pathways leads to strong effects on developmental features and on disease resistance to pathogens. Finally, we will discuss the potential of manipulating hormone homeostasis/signaling to improve crop resistance to pathogen.

## HORMONE REGULATORY NETWORKS IN DISEASE RESISTANCE

### AUXINS

Auxins are a group of molecules including IAA (indole-3-acetic acid) that regulate many aspects of plant development, such as apical dominance, root gravitropism, root hair, lateral root, leaf, and flower formation, and plant vasculature development (Kieffer et al., 2010; Swarup and Péret, 2012). Both direct and indirect effects of auxins on the regulation of pathogen resistance responses in plants have been described (Kazan and Manners, 2009). Indirect effects may be caused by auxins regulation of development-associated processes, such as cell wall architecture, root morphology, and stomata pattern. For example, treatment of rice with IAA impaired the resistance to *Xanthomonas oryzae* pv. *oryzae* probably as a consequence of the activation of the biosynthesis of cell wall-associated expansins that lead to cell wall loosening, which facilitates pathogen growth (Ding et al., 2008).

Auxins can negatively impact plant defense by interfering with other hormone signaling pathways or with PTI (Robert-Seilaniantz et al., 2011a). The bacterial PAMP flg22, a peptide from flagellin protein (Boller and Felix, 2009; Pel and Pieterse, 2012), induces an *Arabidopsis* microRNA (miR393), which negatively regulates the mRNA levels of auxins receptors TIR1 (transport inhibitor response 1), AFB2 (auxin signaling F-box 2), and AFB3. Thus, the flg22-triggered suppression of auxin signaling leads to increased resistance to the bacterium *Pseudomonas syringae* pv. *tomato* DC3000 (*Pst*DC3000) and also to the oomycete *Hyaloperonospora arabidopsidis* (Navarro et al., 2006; Robert-Seilaniantz et al., 2011b). The flg22-induced resistance to these biotrophic pathogens was explained by the observed induction of the SA signaling pathway. Supporting this hypothesis, it was found independently that treatment of *Arabidopsis* leaves with flg22 induces SA accumulation (Tsuda et al., 2008).

In *Arabidopsis*, SA treatment stabilizes the Aux/IAA proteins, leading to down-regulation of the expression of auxin-related genes. Moreover, the enhanced susceptibility to *P. syringae* pv. *maculicola* 4326 (*Psm*4326) of plants expressing the *NahG* gene (encoding a bacterial salicylate hydroxylase that degrades SA) is partially reverted by the *axr2-1* mutation, that disrupts auxin signaling, further indicating that auxin signaling is part of the SA-induced resistance signaling pathway (Wang et al., 2007). Interaction between SA and auxins was further clarified by the characterization of the regulatory pattern of *GH3.5* gene, which is involved in auxin homeostasis in *Arabidopsis* plants. Lines overexpressing *GH3.5* have lower levels of Aux/IAA proteins, overexpression of SA signaling pathway and enhanced resistance to *P. syringae* (Park et al., 2007). Moreover, these transgenic lines also displayed enhanced resistance to abiotic stress and induction of the ABA regulatory pathway (Park et al., 2007).

The conjugated auxin–aspartic acid (IAA–Asp) has been recently reported to play a key role in regulating resistance to the necrotrophic fungus *Botrytis cinerea* and *Pst*DC3000. In *Arabidopsis*, tomato, and *Nicotiana benthamiana* infected with these pathogens there is an enhanced expression of *GH3.2* and *GH3.4* genes, which encode two enzymes required for conjugation of auxins with Asp. Thus, upon pathogen infection, accumulation of IAA–Asp takes place, promoting the development of disease symptoms in infected plants (Gonzalez-Lamothe et al., 2012). The negative effects of auxins on the activation of plant resistance is further supported by the observed enhanced susceptibility of auxin-treated rice to *X. oryzae* (Ding et al., 2008) and of auxin-treated *Arabidopsis* to *Pst*DC3000 (Navarro et al., 2006) and *Fusarium culmorum* (Petti et al., 2012). Disruption of auxin signaling in *Arabidopsis* mutants, such as *axr1*, *axr2*, and *axr3*, leads to enhanced resistance to *F. oxysporum* (Kidd et al., 2011). Nevertheless, auxins have also been shown to positively regulate *Arabidopsis* immunity as *axr2-1* and *axr1-1* mutants were more susceptible than wild-type plants to the necrotrophic fungi *B. cinerea* and *Plectosphaerella cucumerina* (Llorente et al., 2008).

One of the biosynthetic pathways of auxins is partially shared with those required for the biosynthesis of tryptophan-derived antimicrobials, such as indole glucosinolates and camalexin. This might lead to competition for the biosynthetic precursor of auxin and antimicrobials (Barlier et al., 2000; Grubb and Abel, 2006). The recently characterized *Arabidopsis* *wat1* (*walls are thin1*) mutant exhibits specific enhanced resistance to vascular pathogens such as *Ralstonia solanacearum*. This response was associated to a misregulation of tryptophan derivatives (i.e., lower levels of auxin and indole glucosinolates) specifically in roots, resulting in enhanced levels of SA which is, like tryptophan, a chorismate-derivative (Denancé et al., 2013). Collectively, these data demonstrate that auxins play a central role in balancing plant resistance responses.

### ABSCISIC ACID

Abscisic acid is an isoprenoid compound that regulates developmental processes, such as seed development, desiccation, and dormancy (Wasilewska et al., 2008). In addition, the function of ABA as a regulator of abiotic stress has been thoroughly described (Shinozaki and Yamaguchi-Shinozaki, 2007). ABA has also emerged as a complex modulator of plant defense responses (Asselbergh et al., 2008; Feng et al., 2012; Sánchez-Vallet et al., 2012). ABA can function as a positive or a negative regulator of plant defense depending on the plant–pathogen interaction analyzed (Mauch-Mani and Mauch, 2005; Asselbergh et al., 2008; Ton et al., 2009). ABA-impaired (biosynthesis or signaling) mutants in tomato (*sitiens*) and *Arabidopsis* (*abi1-1*, *abi2-1*, *aba1-6*, *aba2-12*, *aao3-2*, and *pyr1pyl1pyl2pyl4*) were shown to overexpress defensive-signaling pathways, leading to enhanced resistance to different pathogens such as *B. cinerea*, *P. syringae*, *F. oxysporum*, *Plectosphaerella* *cucumerina*, and *Hyaloperonospora parasitica* (Audenaert et al., 2002; Mohr and Cahill, 2003; de Torres-Zabala et al., 2007; de Torres Zabala et al., 2009; Garcia-Andrade et al., 2011; Sánchez-Vallet et al., 2012). Negative interactions of ABA with the major hormones involved in plant defense response (SA, JA, and ET) have been described by means of exogenous hormone treatments (Yasuda et al., 2008; de Torres Zabala et al., 2009; Sánchez-Vallet et al., 2012). For instance, almost 65% of the up-regulated genes and 30% of the down-regulated genes in *aba1-6* mutant were found to be up- or down-regulated by either ET, JA, or SA treatment (Sánchez-Vallet et al., 2012). Remarkably, these genes constitutively up-/down-regulated in *aba1-6* mutant were differentially expressed in *Arabidopsis* wild-type plants inoculated with *Plectosphaerella cucumerina*, indicating that they form part of the defensive responses activated upon pathogen infection (Sánchez-Vallet et al., 2012). In addition, ABA plays a direct role in regulating R (resistance) protein activity. ABA and exposition of plants to high temperature both reduce the nuclear accumulation of SNC1 (suppressor of *npr1-1*, constitutive1) and RPS4 (resistant to *Pseudomonas syringae* 4) compromising disease resistance to *P. syringae* (Mang et al., 2012).

Abscisic acid can also positively regulate the resistance to some pathogens, such as *Alternaria brassicicola*, *R. solanacearum*, and *Pythium irregulare*, as ABA-deficient and-insensitive mutants (*abi1-1*, *abi2-1*, *abi4-1*, *aba1-6*, *aba2-12*, *aao3-2*, and *npq2-1*) were found to be more susceptible than wild-type plants to these pathogens (Adie et al., 2007; Hernandez-Blanco et al., 2007; Flors et al., 2008; Garcia-Andrade et al., 2011). In *Arabidopsis*, ABA has been shown to be required for JA biosynthesis that is essential for resistance to *Pythium irregulare* (Adie et al., 2007). This contrasts with the negative interaction of ABA- and JA-signaling in the modulation of *Arabidopsis* resistance to the necrotrophic fungus *Plectosphaerella cucumerina* (Sánchez-Vallet et al., 2012). Similarly, although ABA and SA have been shown to function antagonistically in the control of the resistance to some pathogens, they trigger stomata closure to avoid penetration of the bacteria *P. syringae* in *Arabidopsis* (Melotto et al., 2006). Plant treatment with flg22 is known to interfere with ABA signaling to induce stomata closure. The ABA- or flg22-induced stomata closure are impaired in lines overexpressing HSC70-1 (heat shock cognate70-1) and mutants in HSP90 (heat shock protein90; Clément et al., 2011), resulting in an increased susceptibility to both virulent and avirulent strains of *P. syringae* (Hubert et al., 2003; Takahashi et al., 2003; Noël et al., 2007). ABA is a key hormone in *Arabidopsis* response to *R. solanacearum* infection, as 40% of the genes up-regulated during the development of wilting symptoms were related to ABA, including those encoding proteins for ABA biosynthesis [i.e., 9-*cis*-epoxycarotenoid dioxygenase3 (NCED3)] or signaling [i.e., ABA-insensitive1 (ABI1) and ABI5; Hu et al., 2008]. More recently, it has been shown that pre-inoculation of *Arabidopsis* with an avirulent strain of *R. solanacearum* activates plant resistance to virulent isolates of this bacterium, and this resistance was correlated with the enhanced expression of ABA-related genes that resulted in a hostile environment for the infection development. These results suggest that ABA may be used in biological control of bacterial wilt caused by *R. solanacearum* (Feng et al., 2012).

### SALICYLIC ACID

The function of SA in activating resistance against pathogens has been thoroughly described. In *Arabidopsis*, SA is synthesized from chorismate (a precursor of tryptophan and, consequently, of auxins) *via* two pathways, either through phenylalanine or through isochorismate (reviewed in Vlot et al., 2009). This second pathway, in which SID2/ICS1 (salicylic acid induction deficient 2/isochorismate synthase 1) is involved, is activated upon pathogen infection, such as *Erysiphe* or *P. syringae*, and after plant recognition of pathogen effectors or PAMPs (Tsuda et al., 2008; Vlot et al., 2009). Deficiency of SA biosynthesis in *sid2-1* mutant leads to reduced resistance response in *Arabidopsis* plants (Nawrath and Métraux, 1999). SA is a regulator of plant resistance to biotrophic and hemibiotrophic pathogens, such as *Hyaloperonospora arabidopsidis* and *P. syringae*, and it also regulates systemic acquired resistance (SAR), a well-studied type of induced resistance (Glazebrook, 2005). In addition, SA is a central regulator of immunity. It interacts with other signaling pathways (e.g., ET and JA pathways), as a strategy to induce the proper resistance responses and to reduce the associated fitness costs (Vlot et al., 2009; Thaler et al., 2012).

NPR1 (non-expressor of PR genes 1), a well-known central player in SA signaling (Cao et al., 1997), and NPR3 and NPR4 proteins have been recently described as SA receptors (Fu et al., 2012; Wu et al., 2012). NPR1 localizes at the cytosol as an oligomer, and in the presence of SA, redox changes occurs in NPR1 that lead to the dissociation of NPR1 complex and to the translocation of the corresponding monomers to the nucleus. There, NPR1 protein activates the transcription of defensive genes, such as *PR* (*pathogenesis-related protein*), by interacting with TGA (TGACG sequence-specific binding protein) transcription factors (Dong, 2004; Tada et al., 2008; Robert-Seilaniantz et al., 2011a). In *Arabidopsis*, EDS1 (enhanced disease susceptibility 1) is a major node required both for SA-dependent basal resistance against virulent pathogens and for the activation of the ETI mediated by the TIR–NB–LRR (Toll-interleukin receptor domain–nucleotide binding domain–leucine rich repeat) resistance proteins (Parker et al., 1996; Falk et al., 1999). EDS1 protein is present in distinct pools at nuclei and cytoplasm, and these two EDS1 locations are required for a complete immune response (Garcia et al., 2010). Several EDS1 interactors have been identified, including PAD4 (phytoalexin deficient4), RPS4, RPS6, SAG101 (senescence-associated gene101), SRFR1 (suppressor of RPS4-RLD1), and SNC1 (Feys et al., 2001, 2005; Bhattacharjee et al., 2011; Heidrich et al., 2011; Rietz et al., 2011). The EDS1–PAD4 complex is necessary for basal resistance and activation of SA-defense response (Rietz et al., 2011). Indeed mutations in EDS1 and PAD4 lead to reduce resistance to pathogens such as *Hyaloperonospora parasitica* and deficiency of the SA signaling pathway (Parker et al., 1996; Falk et al., 1999; Jirage et al., 1999). Transcriptional regulation of SA-defensive genes is also mediated by HDA19 (histone deacetylase19) that repressed SA-mediated basal defense to *Pst*DC3000 (Choi et al., 2012). Up-regulation of SA marker genes (*PR1*, *PR2*, *ICS1*, *EDS1*, *PAD4*) and over-accumulation of SA take place in *hda19* mutant, which correlates with its enhanced resistance phenotype to *Pst*DC3000 pathogenic bacteria. Indeed, HDA19 targets *PR1* and *PR2* promoters to regulate gene expression. The mutation *hda19* causes hyper-acetylation of histones in the promoters of *PR* genes and priming of SA-associated plant defense (Choi et al., 2012).

Negative crosstalk between SA and JA signaling pathways has been thoroughly described (Gimenez-Ibanez and Solano, 2013). For example, *WRKY33*, a positive regulator of JA-related genes, is a repressor of the SA pathway. In the *wrky33* mutant there is an enhanced expression of several SA-regulated genes (*SID2*/*ICS1*, *EDS5*/*SID1*, *PAD4*, *EDS1*, *NIMIN1*, *PR1*, *PR2*, *PR3*) and increased accumulation of SA levels. In turn, SA induction contributes to down-regulate JA-signaling, and to increase the susceptibility of *wrky33* plants to necrotrophic fungi (Birkenbihl et al., 2012; Sánchez-Vallet et al., 2012). NPR1 is a regulator of SA-mediated suppression of the JA/ET signaling pathway, as revealed using *npr1* mutant (Spoel et al., 2003). The *Arabidopsis* mediator subunit 16 (MED16) was recently described to be a positive regulator of SA-induced defense response and a negative regulator of JA/ET signaling pathway (Zhang et al., 2012). The negative crosstalk between SA and JA is exploited by *P. syringae* strains producing the phytotoxin coronatine (COR), an structural mimic of the active JA-Ile, to suppress SA signaling (Uppalapati et al., 2005; Zheng et al., 2012). *P. syringae* strains impaired in production of COR have reduced virulence on *Arabidopsis* wild-type plants but not on SA-deficient lines (e.g., *sid2* and *NahG*; Brooks et al., 2005). In a search for *Arabidopsis* mutants in which the virulence of COR-deficient *Pst*DC3000 mutant was recovered, several *scord* (*susceptible to coronatine-deficient* *Pst*DC3000) mutants were found to be defective in SA signaling (Zeng et al., 2011). For instance, *scord3* mutant plants are impaired in EDS5/SID1, a key protein required for SA biosynthesis, and consequently it has reduced SA levels compared with wild-type plants (Zeng et al., 2011), further corroborating the role of SA in resistance to pathogens.

## DECOY STRATEGIES OF PATHOGENS: MANIPULATION OF THE HOST HORMONE MACHINERY

### PATHOGENS PRODUCE AND DEGRADE HORMONES

#### Auxins

Many pathogenic microbes and plant growth promoting rhizobacteria have evolved complete pathways for auxin biosynthesis with tryptophan as the main precursor (Spaepen et al., 2007). Auxin-producing phytopathogenic bacteria are mostly, but not exclusively, gall-inducing microbes. They include, for instance, *Agrobacterium tumefaciens* (Liu and Nester, 2006), *Agrobacterium rhizogenes *(Gaudin and Jouanin, 1995), *Erwinia chrysanthemi* (Yang et al., 2007), *Erwinia herbicola* (Brandl and Lindow, 1998), *Pseudomonas fluorescens* (Suzuki et al., 2003), *P. putida* (Leveau and Lindow, 2005), *Pseudomonas savastanoi* (Glickmann et al., 1998), *P. syringae *(Glickmann et al., 1998), *R. solanacearum* (Sequeira and Williams, 1964; Valls et al., 2006), and *Rhodococcus fascians* (Vandeputte et al., 2005). In *R. solanacearum*, auxin biosynthesis is governed by HrpG, a major regulator of bacterial virulence and response to metabolic signals (Valls et al., 2006). In *Agrobacterium tumefaciens*, two genes required for conversion of tryptophan to auxin are localized on the T-DNA region of the Ti plasmid injected into plant cells. Auxin biosynthesis is necessary for tumor gall formation and for pathogenicity of *Agrobacterium* (Lee et al., 2009): auxins negatively regulate the expression of genes necessary for the transfer of *Agrobacterium* T-DNA in plants and also inhibit the growth of several bacterial species *in vitro* (Liu and Nester, 2006).

Auxin biosynthesis in fungal pathogens seems to be limited to a few species. In *Ustilago maydis*, *U. esculenta*, and *U. scitaminea* auxin is produced (Chung and Tzeng, 2004; Reineke et al., 2008). In this case, auxin does not seem to be required for *U. maydis*-induced tumor formation or for pathogenicity, as a mutant defective in four genes encoding key auxin biosynthetic enzymes was compromised in auxin levels but not in tumor formation (Reineke et al., 2008). Additionally, other fungi have enzymatic tools to produce auxins, such as *Colletotrichum gloeosporioides* f. sp. *aeschynomene*, *Colletotrichum* *acutatum*, and *F. proliferatum* (Robinson et al., 1998; Chung et al., 2003; Maor et al., 2004; Tsavkelova et al., 2012). Nevertheless, the production of auxins by fungal pathogens has not been clearly demonstrated to be a virulent factor that favors plant colonization.

#### Abscisic acid

Several fungal species produce ABA, including *B. cinerea*, *Rhizoctonia solani*, *Ceratocystis fimbriata*, and *Rhizopus nigricans* (Dörffling et al., 1984; Inomata et al., 2004a). ABA biosynthesis by *B. cinerea* requires a cluster of four genes, *BcABA1* to *BcABA4* (Hirai et al., 2000; Inomata et al., 2004b; Siewers et al., 2004, 2006). Unlike plants, fungi, such as *B. cinerea* and *Cercospora* sp., use the mevalonate pathway to produce ABA (Hirai et al., 2000; Inomata et al., 2004a). The role of ABA as a *B. cinerea* virulence factor has not been fully demonstrated, but several published data support this hypothesis: (i) ABA biosynthesis in the fungus is stimulated by the host plant (Kettner and Därffling, 1995); (ii) exogenous treatment with ABA increased disease symptoms caused by the fungus on roses (Shaul et al., 1996); and (iii) ABA contributes to susceptibility to *B. cinerea* and other pathogens by suppressing defense responses in plants (Audenaert et al., 2002; Sánchez-Vallet et al., 2012).

#### Salicylic acid

Although SA biosynthesis has not been described in plant pathogens, it is known that some plant-associated bacteria can degrade salicylate. Indeed, the enzyme salicylate hydroxylase (NahG), that catalyzes the formation of catechol from salicylate, has been identified in various bacteria, such as *P. putida* and *P. fluorescens* (You et al., 1991; Chung et al., 2001).

### PATHOGEN EFFECTORS INTERFERE WITH HORMONE SIGNALING IN PLANTS

**FIGURE 1:**
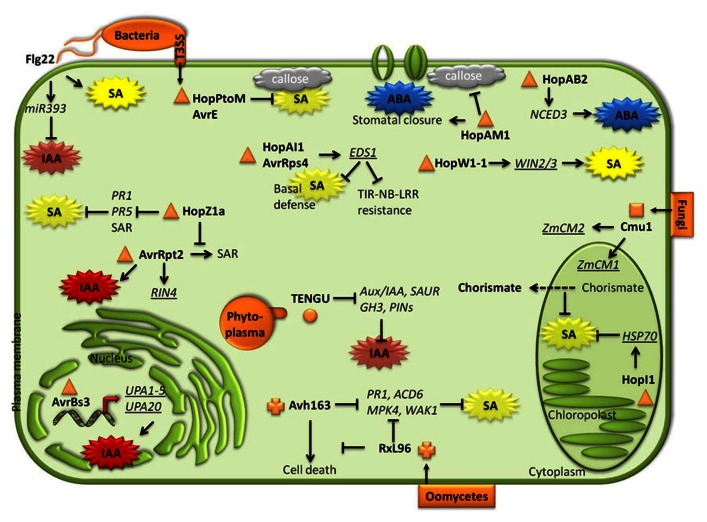
**Decoy strategies elaborated by pathogens and pests to interfere with plant hormone biosynthesis/signaling pathways**. Phytopathogenic bacteria, phytoplasmas, fungi, and oomycetes secrete various effectors inside plant cells during infectious process. Once in the host cells, some effectors specifically bind to (underlined), induce and/or decrease (arrows/crossed lines) target gene expression or protein activity. Consequently, ABA-, SA-, or Auxin-mediated defense mechanisms are activated/repressed.

**FIGURE 2:**
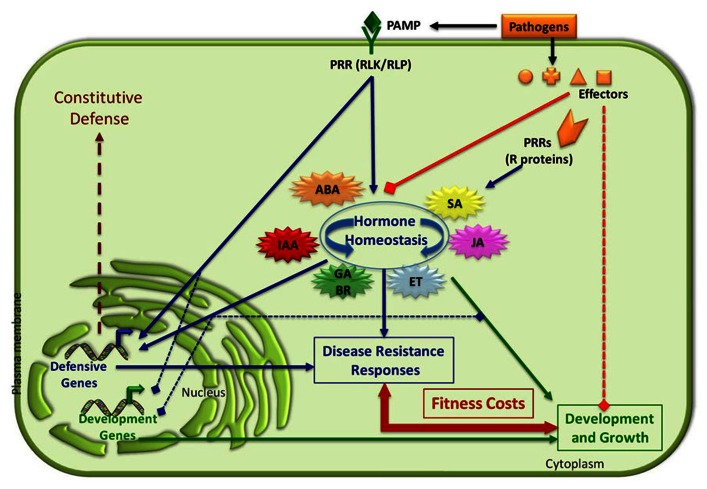
**Balancing plant immune responses and fitness costs**. Plant disease resistance responses are induced upon recognition of PAMPs/effectors from pathogens and pests by plant PRR proteins. This recognition modulates plant hormonal homeostasis and transcriptional reprograming of defensive genes. The activation of these inducible resistance responses (PTI and/or ETI) negatively regulates the expression of developmental-associated genes impacting on plant fitness costs. Effectors from pathogens interfere with hormonal balance and the activation of PTI and ETI. Pathogens can also negatively impact plant growth and developmental-associated processes (transcriptional expression of genes, negative regulation of signaling pathways, etc.; see text for details). Positive and negative interactions are indicated by arrows and squares, respectively. GA, gibberellic acid; BR, brassinosteroids.

Effectors are proteins secreted by pathogens during infection to deregulate host immune responses. One common strategy implemented by effectors is the manipulation of the homeostasis of plant phytohormones, resulting in deactivation of the appropriate defense response (Robert-Seilaniantz et al., 2007; Bari and Jones, 2009; **Figures 1** and **2**).

#### Bacteria and phytoplasma

In addition to the common example of the phytotoxin COR produced by *P. syringae* strains to manipulate the plant hormonal balance (Zheng et al., 2012), many phytopathogenic bacteria have developed large repertoires of type III effectors (T3E) which are necessarily injected through the syringe-like type III secretion system inside plant cells to deregulate plant immunity (**Figure 1**; Jones and Dangl, 2006; Boller and He, 2009; Büttner and He, 2009). The roles of bacterial effectors in plant immunity have been extensively reviewed elsewhere (Cui et al., 2009; Rivas and Genin, 2011; Deslandes and Rivas, 2012; Dou and Zhou, 2012; Howden and Huitema, 2012). *Xanthomonas* sp. bacteria synthesized TAL (transcription activator-like) effectors, such as AvrBs3 from *X. axonopodis* pv. *vesicatoria* (formerly *X. campestris* pv. *vesicatoria*), that are imported to the plant nuclei where they activate the expression of host target genes (Boch et al., 2009; Moscou and Bogdanove, 2009; Bogdanove et al., 2011). Five targets, designed as *up-regulated by AvrBs3 1 to 5* (*UPA1–5*), are auxin-induced genes members of the SAUR (small auxin up RNA) family (Marois et al., 2002). Additionally, induction of the TAL target *UPA20* provokes cell hypertrophy, a feature which is characteristic of auxin accumulation (Kay et al., 2007). Auxin is a susceptibility factor in *Arabidopsis* plants infected with *Pst*DC3000, and consequently, auxin was hypothesized to be a potential target for bacterial effectors. Thus, the cysteine protease bacterial effector AvrRpt2 triggers auxin signaling pathway to enhance bacterial virulence in *Arabidopsis* lines lacking the resistance gene that normally recognizes this T3E. Transgenic plants expressing AvrRpt2 accumulated higher auxin levels and showed a constitutive activation of the auxin signaling pathway. Additionally, auxin levels in *Arabidopsis* leaves inoculated with *Pst*DC3000*avrRpt2* were higher than those in plants infected with *Pst*DC3000 (Chen et al., 2007), indicating that AvrRpt2 modulates auxin pathway to enhance bacterial virulence, but this effect was found to be independent of SA (Chen et al., 2004). Auxin signaling seems to be a preferential target of phytoplasmas, some bacteria-like, obligate plant pathogens belonging to the class of Mollicutes that require sap-feeding insect herbivores as vectors for transmission to plants (Sugio et al., 2011). Indeed, TENGU (tengu-su inducer) is an effector of *Candidatus* phytoplasma asteris that, when expressed in *Arabidopsis* transgenic lines, causes dwarfism and abnormal reproductive organogenesis and flower sterility. These phenotypes, which are similar to the disease symptoms provoked by the phytoplasma, have been associated to alterations in hormone balance. Microarray analysis of transgenic *Arabidopsis* plants expressing TENGU demonstrated that many auxin-related genes were down-regulated, including genes of the *Aux/IAA*, *SAUR*, *GH3*, and *PIN* families (Hoshi et al., 2009). Thus, TENGU effector could interfere with auxin signaling in plants. 

Several *P. syringae* effectors target SA. HopPtoM and AvrE are repressors of SA-dependent callose deposition but do not affect SA-responsive genes in *Arabidopsis* infected leaves (DebRoy et al., 2004). The effector HopI1 (previously named HopPmaI), that is essential for the virulence of *P. syringae* pv. *maculicola* (*Pma*) in *Arabidopsis*, *N. benthamiana*, and *N. tabacum*, has been found to be a modulator of SA-mediated defense responses. Indeed, the expression of HopI1 in *Arabidopsis acd6-1* (*accelerated death6-1*) mutant reduces the enhanced SA levels and the constitutive induction of defense responses characteristic of this mutant (Jelenska et al., 2007). Another effector, HopZ1a, a cysteine protease from *P. syringae* that interferes with SA signaling, is able to suppress *Pst*DC3000-induced expression of *PR1* and *PR5* and the SAR induced either by *Pst*DC3000 (virulent) or *Pst*DC3000*avrRpt2* (avirulent) pathogens (Macho et al., 2010). Thus, HopZ1a contributes to *Pst* virulence by suppressing SA-mediated defenses that takes place during ETI induced by other effectors such as AvrRpt2. EDS1, a key regulatory node of basal and induced resistance, is also targeted by bacterial pathogen effectors. AvrRps4 and HopA1, two *Pst*DC3000 effectors, bind to EDS1 interfering with the interaction between EDS1 and TIR–NB–LRR resistance proteins, and consequently preventing the activation of the immune response (Bhattacharjee et al., 2011; Heidrich et al., 2011). In contrast to other effectors, HopW1-1, that forms part of the T3E repertoire of *Pma*, but not of that of *Pst*DC3000 (Guttman et al., 2002), induces resistance in the Ws accession of *Arabidopsis* to *Pma* (Lee et al., 2008). This effect of HopW1-1 on Ws was corroborated by the fact that *Pst*DC3000 strain expressing HopW1-1 has reduced growth and caused weak disease symptoms in the Ws plants. In a yeast two-hybrid screen, three *Arabidopsis* HopW1-1-interacting proteins (WIN2, WIN3) were found to bind to the effector (Lee et al., 2008). The enhanced resistance triggered by HopW1-1 was not caused by activation of a hypersensitive response, but it was dependent on an enhanced accumulation of SA. Indeed, *pad4* mutants were almost completely compromised in their resistance response to HopW1-1.

HopAM1 contributes to *P. syringae* virulence by manipulating ABA-mediated responses in plants: it enhances stomata closure, suppresses infection-triggered callose deposition, and inhibits seed germination. Remarkably, HopAM1 increased *P. syringae* virulence on *Arabidopsis* plants grown under water-stressed conditions (Goel et al., 2008). *Arabidopsis* lines expressing HopAM1 showed enhanced colonization by the avirulent *Pst*DC3000 *hrcC*^-^ mutant, impaired in T3SS, and did not develop callose-rich papillae that are normally induced by *hrcC*^-^ strain in wild-type plants (Goel et al., 2008). An effector of *P. syringae* pv. *phaseolicola*, HopAB2, promotes virulence on *Arabidopsis* and bean plants, and suppresses basal resistance to *Pst*DC3000 *hrpA*^-^, a mutant compromised in T3SS (de Torres et al., 2006). Expression of HopAB2 in *Arabidopsis* plants induces the expression of *NCED3*, resulting in enhanced biosynthesis of ABA, which interferes with the accumulation of SA levels and the activation of SA-mediated resistance (de Torres-Zabala et al., 2007). Thus, HopAM1 and HopAB2 are suppressors of defense mechanisms by enhancing ABA responses and promoting disease susceptibility in plants.

#### Filamentous pathogens: oomycetes and fungi

Oomycete genomes contain a class of cytoplasmic proteins known as RXLRs that contain a conserved RXLR amino acid motif (arginine, any amino acid, leucine, arginine; Rehmany et al., 2005; Morgan and Kamoun, 2007). Two effectors from this class, HaRxL96 from *Hyaloperonospora arabidopsidis*, the causal agent of downy mildew on *Arabidopsis*, and its ortholog PsAvh163 from *Phytophthora sojae*, which causes soybean rot disease, interfere with plant immunity (Anderson et al., 2012). Remarkably, *Arabidopsis* plants expressing HaRxL96 or PsAvh163 became more susceptible to virulent and avirulent pathogens, indicating that these effectors repress basal resistance and ETI. In fact, the induction of SA-defensive genes, but not SA biosynthesis, that take places upon infection with avirulent strains of *Hyaloperonospora arabidopsidis*, was suppressed in the transgenic lines expressing HaRxL96 or PsAvh163, indicating that these effectors interfere with SA signaling to trigger plant susceptibility to oomycetes (Anderson et al., 2012).

Filamentous extracellular or obligate fungal pathogens secrete effectors via hyphae or haustoria (Stergiopoulos and de Wit, 2009; de Jonge et al., 2011). *U. maydis* is a basidiomycete fungus that causes smut disease on maize and its relative teosinte (Brefort et al., 2009; Djamei and Kahmann, 2012). Maize infection by *U. maydis* results in the repression of SA-associated *PR1* defense gene expression during the early biotrophic phase of the interaction, while auxin production in the host is induced later during tumor formation (Doehlemann et al., 2008). One of the most highly expressed genes of *U. maydis* during plant colonization is the Cmu1 effector, a chorismate mutase protein (Skibbe et al., 2010). Cmu1 is required for full virulence since the induction of tumors is significantly reduced in a *U. maydis* *cmu1* mutant (Djamei et al., 2011). Once inside plant cells, Cmu1 is localized in the cytoplasm, the nucleus and guard cells and it is spread to neighbor cells through plasmodesmata. A yeast two-hybrid analysis showed that Cmu1 interacts with two maize chorismate mutases, ZmCm1 and ZmCm2, which are found in plastids and cytoplasm in plants, respectively. Interestingly, SA levels were higher in maize inoculated with a *cmu1* mutant than with a wild-type strain, resulting in an increased resistance of the mutant to *U. maydis*. It was hypothesized that Cmu1 could act together with ZmCm2 in the plant cytoplasm to enhance the flow of the SA-precursor chorismate from the plastid (where SA biosynthesis takes place) to the cytosol. Consequently, in plastids, less chorismate would be available for SA biosynthesis (Djamei et al., 2011). These results indicate that SA biosynthesis pathway of maize is hijacked by *U. maydis* as a mechanism of virulence. Interestingly, such a mechanism was also described for the soybean cyst nematode *Heterodera glycines* and the root-knot nematode *Meloidogyne javanica* (Bekal et al., 2003; Doyle and Lambert, 2003). The virulence factor of *Cladosporium fulvum* Avr2 targets the tomato papain-like cysteine protease (PLCP) RCR3 and *Phytophthora*-inhibited protease 1 (PIP1) in order to deregulate basal immunity. RCR3 and PIP1 are specifically induced by treatment of tomato plants with the SA analog benzothiadiazole (BTH). Therefore, Avr2 seems to interfere with tomato SA signaling pathway (Shabab et al., 2008).

## FITNESS COSTS OF DEFENSE RESPONSES REGULATED BY PHYTOHORMONES

The involvement of many plant growth regulatory phytohormones in the control of plant resistance responses to both biotic and abiotic stresses indicates the existence of a tight interconnection between two physiological processes: development and adaptation to environmental cues. The regulatory potential of the hormone network allows plants to quickly respond to environmental changes and, thus, to use the limited nutrient resources in a cost-efficient manner. This hypothesis is based on the idea that being well-defended (i.e., having strong, pre-existing defensive mechanisms) may not always be the best defensive strategy, most likely because allocation of metabolites and proteins to resistance may constrain other plant physiological processes (Walters and Heil, 2007; Manzaneda et al., 2010; Kempel et al., 2011). In line with this hypothesis, it is generally believed that hormone-induced resistance evolved to save energy under enemy-free conditions, as they will only incur energy costs when these defensive mechanisms are activated upon pathogen infection or insect attack (Walters and Heil, 2007). However, pathogens and pests evolve to get adapted to the continuous exposure to defensive genetic traits (i.e., antibiotic or antideterrent proteins and/or metabolites). Therefore, it is also possible that hormone-induced resistance evolved to slow down the potential adaptation of putative attackers to these biochemical barriers (Walters and Heil, 2007). All these physiological constrains, together with the co-existence of plants with natural attackers, have evolutionary driven the selection of plant innate immune system.

In different plant species there have been characterized mutants or transgenic lines showing constitutive activation of defensive mechanisms and enhanced resistance to particular pathogens. These resistance phenotypes are generally associated with the misregulation of particular hormone signaling pathways (Robert-Seilaniantz et al., 2011a). The characterization of these mutants and transgenic plants has contributed to the identification of the molecular components involved in hormone biosynthesis and signaling pathways, and to the discovery of cross-regulatory nodes among these signaling pathways. Thus, *Arabidopsis* mutants constitutively overexpressing a specific hormone-dependent pathway (SA, ET, JA, ET + JA, etc.) show enhanced resistance to particular type of pathogens (reviewed by Robert-Seilaniantz et al., 2011a; Holeski et al., 2012). However, this enhanced, constitutive resistance negatively impact plant fitness as these mutants have phenotypic alterations such as dwarfism, spontaneous lesions in different organs, accelerated senescence, delayed flowering, sterility, or reduced seed production (for a review, see Robert-Seilaniantz et al., 2011a; Holeski et al., 2012; Thaler et al., 2012). These data indicate that plants have genetic determinants to fine-tune fitness/resistance balance. An example of this fine-tune regulation is represented by the SA receptor NPR3, that is a negative regulator of defensive response during *Arabidopsis* early flower development through its interaction with NPR1 and TGA2. Remarkably, the *nrp3* plants exhibit increased resistance to *P. syringae* infection of immature flowers, but showed reduced fitness in comparison to that of wild-type plants (Shi et al., 2012).

Alteration of a particular hormone signaling pathway generally results in the miss-regulation of other signaling pathways due to the described complex regulatory network that exist among hormones. Thus, the negative cross-regulations among hormone pathways, such as auxin, ABA, and SA described in this review, lead to alterations in the pattern of resistance to natural attackers. That is, enhanced resistance to a particular pathogen (i.e., necrotroph) can be achieved in some of these mutants, but they generally undergo increased susceptibility to a different one (i.e., biotroph; Spoel et al., 2007; Robert-Seilaniantz et al., 2011a). In some particular cases, such as in the ABA-deficient mutant *aba1*, broad spectrum resistance to both necrotrophic and biotrophic pathogen is observed, but this phenotype is also linked to a reduced adaptation of the mutant to abiotic stresses such as drought (Audenaert et al., 2002; de Torres-Zabala et al., 2007; de Torres Zabala et al., 2009; Garcia-Andrade et al., 2011; Sánchez-Vallet et al., 2012). As in nature, plants are exposed to many different biotic agents, but also to abiotic stress, hormone homeostasis is critical in the establishment of appropriate and effective defensive responses of plant against natural attackers and/or abiotic stresses in an ecological context (**Figure 2**). In line with the hypothesis of the critical role of hormones in balancing growth and response to environmental cues, it has been recently demonstrated that brassinosteroids, that control several developmental-associated processes, also modulate the efficiency of PTI in *Arabidopsis* (Belkhadir et al., 2012). The interaction between these two types of environmental stresses (biotic and abiotic) requires a complex adaptive molecular response involving many factors that we are just starting to understand (reviewed in Atkinson and Urwin, 2012).

Expressing constitutive resistance by the modification of hormone homeostasis/signaling encounters the risk of allocating resources to defense in the absence of natural pathogens and of impairing defensive mechanisms against particular natural attackers. One alternative to the constitutive, long-lasting activation of induced resistance is to fine-tune plant resistance mechanism by modulating the “immunological memory” of plants, as it has been described in animals (Conrath, 2011). An interesting phenomenon in this context is the so-called “priming” that is a condition whereby plants that have been subjected to prior attack will respond more quickly or more strongly to a subsequent attack. Given that resources are not committed until the threat returns, priming is thought to be a relatively low-cost mechanism of advancing plant defense (Conrath, 2011). Remarkably, the resistance response in primed plants treated with a low, non-effective concentration of a defensive hormone is also faster and stronger than that in non-primed plants (Conrath, 2011). It has been recently demonstrated the existence of an epigenetic regulation of priming, which explain the lack of significant transcriptional changes in primed plants unless they are exposed to the priming agent/hormone (Luna et al., 2012; Slaughter et al., 2012). The genetic control of priming shows similarities to the genetic mechanisms that regulate transgenerational defense induction in plants, such as the SA-dependent SAR and the inherited JA-dependent defense (Holeski et al., 2012; Thaler et al., 2012; Zheng et al., 2012). Similarly, transgenerational priming has been also described (Luna et al., 2012; Slaughter et al., 2012). All these epigenetically inherited changes in defense can strongly alter plant responses to jasmonate and salicylate in offspring and therefore might negatively impact plant resistance to particular type of pathogens (Latzel et al., 2012; Luna et al., 2012).

Though all the published data clearly indicate a fitness cost associated to the constitutive activation of hormone-mediated resistance mechanisms, it must be considered that these experiments were generally performed under laboratory conditions, without nutrient limitations and ecological constraints (i.e., plants were infected with just one pathogen). Long-term experiments with model and crop plants under field conditions should be done to determine the potential use of hormone-mediated resistance in crop protection, as these experiments will provide information on the hormone-mediated effectiveness of disease control, but also on plant trade-offs and changes in the population structure of pathogens and pests. Also, a better understanding of the molecular and genetic mechanisms regulating hormone-mediated resistance would be required to successfully manipulate hormone homeostasis/signaling and improve crop resistance to pathogens.

## Conflict of Interest Statement

The authors declare that the research was conducted in the absence of any commercial or financial relationships that could be construed as a potential conflict of interest.
